# Functional and Aesthetic Assessment of Facial Reanimation with Free Gracilis Flap Transfer in Chronic Facial Paralysis

**DOI:** 10.1055/s-0045-1814753

**Published:** 2026-02-03

**Authors:** Paula Weinberger-Forische, Kenzo Alejandro Fukumoto-Inukai, Damián Palafox, Alejandra Nicole Llamas-Ostos, Miguel Angel Dominguez-Varela, Lucio Alejandro Santos-Moyron, Alexander Cárdenas-Mejía

**Affiliations:** 1 Plastic and Reconstructive Surgery Department, Hospital General “Dr. Manuel Gea Gonzalez,” Mexico City, Mexico; 2 Plastic and Reconstructive Surgery Department, Facial Paralysis and Peripheral Nerve Surgery Clinic, Hospital General “Dr. Manuel Gea González,” Mexico City, Mexico

**Keywords:** facial paralysis, gracilis muscle flap, facial reanimation

## Abstract

**Introduction:**

Chronic facial paralysis severely compromises facial symmetry and function. The free gracilis muscle flap has become the gold standard for dynamic facial reanimation. This study aims to evaluate the functional and aesthetic outcomes of gracilis free muscle flap procedures performed at the Facial Paralysis Clinic of Hospital General “Dr. Manuel Gea González,” using the Terzis Functional Grading System and the eFACE (Electronic Facial Paralysis Assessment) digital assessment tool.

**Materials and Methods:**

A retrospective, cross-sectional, and analytical study was conducted on 52 patients who underwent free gracilis muscle flap surgery for facial reanimation between 2018 and 2023. Inclusion criteria were chronic facial paralysis and complete pre- and postoperative assessments using the Terzis grading system. In unilateral cases, the eFACE scale was also used. Functional and aesthetic outcomes were analyzed using paired Student's *t* -tests.

**Results:**

Of the 52 procedures, 62% involved unilateral paralysis. The most common etiology was classic Moebius syndrome (35%). Significant functional improvement was observed on the Terzis scale, with mean scores increasing from 1.1 to 3.1 postoperatively ( *p* < 0.01). In unilateral cases, the eFACE scale demonstrated significant improvements in static symmetry, dynamic movement, midface/smile, and lower face/neck domains. No significant changes were observed in synkinesis or periocular function. Postoperatively, 52% of patients achieved good outcomes (group IV), and 86% of procedures were free of complications.

**Conclusion:**

The free gracilis muscle flap significantly improves facial function and aesthetic in patients with chronic facial paralysis. These findings validate its effectiveness and highlight the value of objective assessment tools for outcome measurement and future research.

## Introduction

Facial paralysis is the partial or complete loss of voluntary facial muscle movement due to facial nerve dysfunction, most commonly affecting one side of the face, although bilateral cases can occur. 1 It is classified by duration as acute (≤ 72 hours), subacute (72 hours–12 to 18 months), or chronic (> 12–18 months). 2 Etiologies are broadly categorized as central or peripheral. Central causes such as stroke, tumors, or even trauma can affect the contralateral lower face, sparing the forehead and eyelid. 3 In contrast, peripheral paralysis affects the entire ipsilateral hemiface, with Bell's palsy (idiopathic) being the most prevalent etiology, accounting for 50 to 70% of cases. 4

At our institution, the Plastic and Reconstructive Surgery Department of Hospital General “Dr. Manuel Gea González” in Mexico City, congenital facial paralysis, predominantly due to Moebius syndrome represents 42% of cases, followed by iatrogenic causes (29%) and Bell's palsy (16%). 5

Surgical facial reanimation with free functional muscle transfer, particularly the gracilis muscle, is a well-established treatment for chronic facial paralysis. However, standardized outcome evaluation remains a challenge. Both subjective and objective tools are usually employed. Among scales, the Terzis Facial Grading System is widely used, rating smile symmetry and aesthetic outcome from 1 (poor) to 5 (excellent). 6 7 8 This scale is applied in our clinic both preoperatively and postoperatively, and can be used in patients with unilateral or bilateral paralysis.

The eFACE (Electronic Facial Paralysis Assessment) scale is a validated, digital, physician-graded tool incorporating 15 visual analog items across static function, dynamic movement, and synkinesis. 9 It provides numerical and graphical outputs, with strong inter- and intrarater reliability. 9 10 Though designed for unilateral facial paralysis, it has been used in syndromic and congenital cases. A Spanish version has also been validated, showing high reliability and utility among Spanish-speaking clinicians. 11

The outcomes of gracilis free muscle flaps have been reported globally, notably by Terzis et al, who analyzed over 100 free muscle transfers using their own functional and aesthetic classification. 7 In our institution, Cárdenas et al previously reported postoperative improvements using the Terzis scale in patients with Moebius syndrome, increasing from a mean of 1 preoperatively to 3.5 to 4.3 postoperatively. 12 The eFACE scale has also demonstrated postoperative improvements in facial symmetry and function in select studies carried in our institution. 13 14

Despite these findings, there is a lack of comprehensive outcome studies evaluating gracilis free flap reanimation in larger, heterogeneous patient populations. Our institution performs a high volume of these procedures but has not yet published a broad outcome analysis. This study aims to fill that gap by evaluating functional and aesthetic outcomes using the Terzis and eFACE scales. We hypothesize that patients undergoing gracilis free muscle flap reanimation will show a mean improvement of at least 2 points on the Terzis scale postoperatively.

## Materials and Methods

This cross-sectional, observational, analytical, and retrospective study was conducted using medical records of patients with chronic facial paralysis treated at the Facial Paralysis Clinic of the Plastic and Reconstructive Surgery Department at the Hospital General “Dr. Manuel Gea González.” All patients included had undergone facial reanimation using a free gracilis muscle flap. Data were collected from surgical reports, clinical notes, and standardized preoperative and postoperative assessment forms. Functional and aesthetic outcomes were evaluated at baseline and at least 6 months after surgery.

The free gracilis muscle flap was performed unilaterally (right or left) or bilaterally in a single surgical procedure. In cases where the procedure was bilateral and performed in a single operative time, each flap was considered as a separate surgical procedure.

While it is not the primary scope of this article to describe the surgical technique in detail, we herein provide a brief summary of the procedure. Two-stage reconstructions were performed when using a cross-facial nerve graft (CFNG) to provide spontaneous, emotionally driven movement. The first stage involved harvesting and coapting the sural nerve graft to branches of the contralateral facial nerve. The second stage, usually performed 6 to 9 months later, consisted of free gracilis muscle transfer and innervation through the matured CFNG. Techniques are performed as previously described in our cohort of Moebius syndrome patients.

Single-stage procedures were selected when the masseteric nerve was used as the donor nerve, providing strong, immediate neural input for faster reinnervation and muscle contraction. 15 16

Only records that included complete pre- and postoperative evaluations using the Terzis Aesthetic and Functional Grading Scale were included. In cases of unilateral facial paralysis, the eFACE scale was also applied and recorded ( Appendix 1 depicts Terzis Functional and Aesthetic Grading system).

Inclusion criteria were:

- Medical records of patients with unilateral or bilateral chronic facial paralysis.
- Underwent free gracilis muscle flap for facial reanimation.
- Had complete Terzis scale scores before and after surgery.
- For unilateral cases, the availability of eFACE scale scores before and after surgery.

Exclusion criteria were:

- Lack of postoperative rehabilitation after surgery.
- Underwent additional facial reanimation procedures following the free gracilis flap.

Patients who underwent additional facial reanimation procedures following the initial free gracilis muscle transfer were excluded to ensure that postoperative functional and aesthetic outcomes reflected the results of the gracilis flap alone, without confounding influences from subsequent interventions.

Sample size was calculated using the G*Power software (version 3.1). A total of 52 free gracilis muscle flap procedures performed in 52 patients from 2018 to 2023 were included after confirming eligibility according to the inclusion and exclusion criteria. Of these cases, 31 were females and 21 males, with 32 cases being unilateral paralysis and 20 bilateral paralysis. A total of three cases were excluded from the study for not meeting criteria.

The following variables were collected: age, gender, laterality of facial paralysis (unilateral, bilateral), etiology (traumatic, iatrogenic, neoplastic, infectious due to herpes zoster oticus, Bell's palsy [idiopathic], or developmental syndromes such as classic, incomplete, or Moebius-like), duration of paralysis before surgery, donor nerve, side of surgery, number of surgical stages (single- or two-stage), and postoperative complications (hematoma, abscess, flap loss).

Outcome measures included pre- and postoperative scores from the Terzis grading system for all procedures. For unilateral facial paralysis cases, scores from all six domains of the eFACE scale (static function, dynamic function, synkinesis, periocular region, lower face/neck region, and midface/smile) were also analyzed.

Preoperative and postoperative functional and aesthetic evaluations using the Terzis Functional and Aesthetic Grading System and the eFACE scale were conducted by two senior independent evaluators from the Facial Paralysis Clinic. Both evaluators were blinded to each other's assessments, and the mean of both scores was used for statistical analysis to minimize interobserver bias.

Paired Student's *t* -tests were used to compare pre- and postoperative Terzis and eFACE scores. All statistical analyses were performed using SPSS version 25 (IBM Corp., Armonk, New York, United States).

Detailed rehabilitation protocols were not analyzed in this study, as the focus was on surgical outcomes. However, in brief: postoperative rehabilitation consisted of individualized passive and active facial exercises, progressing to neuromuscular retraining once reinnervation was observed. Biofeedback techniques were also employed to enhance motor control and symmetry of facial movements. All rehabilitation protocols were performed by board-certified specialists in rehabilitation. Due to geographic and socioeconomic factors, many patients remained hospitalized for extended periods to ensure supervised therapy, as outpatient follow-up could be inconsistent.

## Results

A total of 52 surgical procedures were included in the study, performed predominantly in female patients (59%), with a mean age of 24.7 years at the time of surgery (± 18.81 years). The clinical characteristics, procedural variations, and postoperative complications associated with free gracilis muscle flap reconstructions are summarized in Tables 1 and 2 .

**Table 1:** Age and duration of facial paralysis at the time of the facial reanimation surgery in the study sample

	Mean	Standard deviation	Minimum	Maximum
Age (y)	24.7	18.81	3	70
Time since onset (mo)	97.3	85.7	24	348

**Table 2:** Clinical characteristics, variations, and complications of free gracilis muscle flap procedures in the study sample

Variable	All cases ( *n* = 52)
Gender	
Female	31 (59%)
Male	21 (41%)
Side of facial paralysis	
Unilateral	32 (62%)
Bilateral	20 (38%)
Etiology of facial paralysis	
Trauma	2 (4%)
Malignancy	17 (33%)
Idiopathic (Bell's paralysis)	7 (13%)
Classic Moebius	18 (35%)
Incomplete Moebius	8 (15%)
Moebius-like	0
Donor nerve	
Spinal + CFNG	1 (2%)
Masseter	20 (38%)
Masseter + CFNG	31 (60%)
Hypoglossal	0
Side of the procedure	
Right	24 (46%)
Left	28 (54%)
Number of surgical stages	
1	12 (23%)
2	40 (77%)
Postsurgical complications	
Hematoma	5 (10%)
Flap loss	2 (4%)
Abscess	0
None	45 (86%)

Abbreviation: CFNG, cross-facial nerve graft.

Unilateral facial paralysis was more frequent, accounting for 62% of cases, with a mean duration of 97.3 months before surgical intervention. The most common etiology was classic Moebius syndrome (35%), followed by neoplastic causes (33%), incomplete Moebius syndrome (15%), idiopathic facial paralysis (13%), and traumatic facial paralysis (4%).

Regarding surgical variations, 54% of the procedures were performed on the left side of the face. The most frequently used donor nerves were the masseteric nerve combined with a CFNG (60%), followed by the masseteric nerve alone (38%) and, less commonly, the spinal accessory nerve with a CFNG (2%). The hypoglossal nerve was not used as a donor nerve in any case. Most procedures (77%) were completed in two surgical stages. Postoperative complications included hematoma occurring in 10% of cases and flap loss in 4%. Of note, 86% of procedures were free of any other major postoperative complications.

Outcomes were evaluated using the Terzis Aesthetic and Functional Grading Scale for all procedures, and the six domains of the eFACE scale were assessed in patients with unilateral facial paralysis, comparing preoperative and postoperative results. As shown in Table 3 , a statistically significant improvement was observed in the Terzis scale scores, as well as in the static, dynamic, midface/smile, and lower face/neck domains of the eFACE scale. No significant differences were detected in the synkinesis or periocular region domains. The mean preoperative Terzis score was 1.1, increasing to 3.1 postoperatively, representing a 2-point improvement.

**Table 3:** Preoperative and postoperative comparison of the mean scores of the Terzis and eFACE scales

	Presurgical mean score (standard deviation)	Postsurgical mean score (standard deviation)	*p-Value*
Terzis grading system	1.1 (0.3)	3.1 (1.2)	< 0.001
eFACE static	57.5 (17.1)	76.5 (16.4)	< 0.001
eFACE dynamic	25.3 (13.0)	49.4 (16.8)	< 0.001
eFACE synkinesis	94.3 (7.5)	95.5 (4.7)	0.163
eFACE periocular	66.1 (14.3)	66.9 (15.1)	0.318
eFACE inferior third and neck	65.3 (4.2)	81.9 (10.3)	< 0.001
eFACE medial third of the face and smile	45.8 (15.3)	78.2 (18.1)	< 0.001

Abbreviation: eFACE, Electronic Facial Paralysis Assessment.

Note: Student's *t* -test.

The normality test of the sample was performed using the Shapiro–Wilk test, with a mean of 0.906 ( *p* < 0.001).

As detailed in Table 4 , before surgery, all patients were classified into Terzis groups I or II. Following reanimation surgery, 52% of patients achieved a good outcome (group IV), while 19.2% achieved a moderate outcome. A poor or bad outcome was observed in 11.5 and 17.3% of cases, respectively.

**Table 4:** Frequency and percentage of Terzis scale grades preoperatively and postoperatively

Group	Terzis grading system	
	Presurgical, *n* (%)	Postsurgical, *n* (%)
I (poor)	45 (86.5)	9 (17.3)
II (fair)	7 (13.5)	6 (11.5)
III (moderate)	0	10 (19.2)
IV (good)	0	27 (52)
V (excellent)	0	0

## Discussion

Facial reanimation with free gracilis muscle transfer is widely regarded as the gold standard for restoring dynamic facial movement in cases of chronic facial paralysis. Its reliable anatomy, favorable functional outcomes, and minimal donor site morbidity make it the procedure of choice across a range of etiologies. In our cohort, we observed a mean improvement of 2 points on the Terzis scale postoperatively, reaffirming the effectiveness of this approach.

These findings are consistent with previously published data. Terzis et al reported improvements from a mean score of 1.6 to 3.0 following gracilis or pectoralis minor muscle transfers. 7 More recent studies have also shown comparable gains. Kim et al demonstrated that double-innervated gracilis flaps produced greater functional recovery (from 1.41 to 3.63). 17 Similarly, Tzafetta et al and Biglioli et al reported postoperative scores up to 4.3, with 50% of patients achieving good outcomes. 18 19 In Mexico, Cárdenas et al achieved a mean postoperative score of 4.33 using a two-staged, double-innervation approach. 12

Despite shared trends, outcomes across studies vary based on several factors. Differences in surgical technique, timing, surgeon expertise, and rehabilitation protocols all influence success. Patient-specific variables—such as age, etiology, and severity—also play a critical role. Our study's inclusion of a heterogeneous patient population, while reflective of real-world clinical practice, likely contributed to outcome variability.

In cases with suboptimal recovery, prolonged denervation time, congenital etiologies (notably Moebius syndrome), and irregular rehabilitation adherence appeared to be associated with poorer outcomes. Optimizing patient selection, standardizing rehabilitation, and considering double-innervated flap techniques may help enhance future results.

## Evaluation Tools

The Terzis scale remains a widely accepted tool due to its simplicity and applicability to both unilateral and bilateral cases. In our study, it was effective in capturing aesthetic and functional changes, particularly in patients with congenital conditions like Moebius syndrome.

Although the eFACE scale provides a more objective, digitized assessment and demonstrated improvements in unilateral paralysis cases, its inability to evaluate bilateral facial function limits its broader applicability. The Terzis scale, despite being subjective and prone to interobserver variability, remains necessary for evaluating bilateral cases, especially where digital alternatives fall short.

## Clinical Implications

Our findings support the broad utility of free gracilis muscle transfer across diverse etiologies, including congenital (e.g., Moebius syndrome) and acquired (e.g., trauma, neoplasm) facial paralysis. The inclusion of both unilateral and bilateral cases underlines the versatility of this technique.

Few studies have assessed outcomes across such a diverse patient population without restricting for surgical technique or diagnosis. Our study adds value by capturing all gracilis procedures performed over a 5-year period at a single, high-volume center. To our knowledge, this is the first such report from Latin America, offering insight into a patient demographic underrepresented in international literature.

Importantly, improvements in Terzis scores likely reflect more than statistical gains—they may translate into meaningful improvements in social interaction, self-image, and quality of life. However, future studies should incorporate patient-reported outcome measures (PROMs) to better quantify these benefits.

## Limitations

Our study is not without limitation. We acknowledge these limitations in an effort to improve transparency and provide the readers and colleagues with a better understanding of our study's strengths and weakness. The retrospective nature of the study limits its ability to control for confounding variables and introduces potential selection bias. The lack of standardized follow-up intervals and rehabilitation protocols may also have influenced results. While we used both the Terzis and eFACE scales, the former is subjective in nature, and the latter is limited to unilateral cases, highlighting a gap in standardized tools for bilateral facial paralysis.

Additionally, the heterogeneity of our cohort complicates subgroup analyses. Future studies should employ prospective designs, standardized surgical protocols, and larger sample sizes to evaluate outcomes by technique, innervation strategy, or etiology.

By excluding patients who required secondary reanimation procedures, we sought to isolate the outcomes of the primary gracilis flap. However, this may have introduced selection bias by excluding cases with poor primary outcomes, which should be considered when interpreting our results.

Postoperative rehabilitation was not standardized across all patients due to geographic, socioeconomic, and logistic factors inherent to our setting, which may have influenced functional outcomes. Future studies should incorporate structured rehabilitation protocols, including biofeedback and neuromuscular retraining, to better assess their impact on recovery.

The integration of advanced technologies—such as three-dimensional motion analysis—and validated PROMs like the Facial Disability Index or FaCE (Facial Clinimetric Evaluation) scale could enhance the assessment of both functional and psychosocial outcomes. Recently, researchers have found promising results using artificial intelligence and machine learning to analyze video footage, with the goal of creating an automated tool to assess facial paralysis. 20

## Conclusion

Facial reanimation by means of free gracilis muscle flap transfer remains a reliable and effective option for facial reanimation in chronic facial paralysis, yielding significant functional and aesthetic improvements across a range of etiologies. Our findings, consistent with international literature, support its broad applicability, including in both unilateral and bilateral cases. This study represents one of the first comprehensive outcome evaluations in a Latin American cohort, contributing valuable regional data. Future prospective studies should incorporate standardized objective measures and patient-reported outcomes to better assess long-term functional recovery and quality of life.

**Appendix 1:** Terzis Functional and Aesthetic Grading system

Group	Grading	Description	Result
I	1 point	Deformity, no contraction	Poor
II	2 points	No symmetry, minimal contraction	Fair
III	3 points	Moderate symmetry and contraction	Moderate
IV	4 points	Symmetry, nearly full contraction	Good
V	5 points	Symmetrical smile with full contraction	Excellent

## References

[1] Lassaletta L Morales-Puebla J M Altuna X Facial paralysis: clinical practice guideline of the Spanish Society of Otolaryngology [in Spanish] Acta Otorrinolaringol Esp (Engl Ed) 2020 71 02 99 118 31097197 10.1016/j.otorri.2018.12.004

[2] Brown S Isaacson B Kutz W Barnett S Rozen S M Facial nerve trauma: clinical evaluation and management strategies Plast Reconstr Surg 2019 143 05 1498 1512 30807496 10.1097/PRS.0000000000005572

[3] Tzou C HJ Rodríguez-Lorenzo A Facial Palsy: Techniques for Reanimation of the Paralyzed Face 1st ed. Vienna, Austria Springer Nature 2021

[4] Aronson S Applebaum S A Kelsey L J Gosain A K Evidence-based practices in facial reanimation surgery Plast Reconstr Surg 2023 152 03 520e 533e 10.1097/PRS.0000000000010539 37647378

[5] Chávez-Serna E Telich-Tarriba J E Altamirano-Arcos C Nahas-Combina L Cárdenas-Mejía A Facial paralysis, etiology and surgical treatment in a tertiary care center in plastic and reconstructive surgery in Mexico Cir Cir 2021 89 06 718 727 34851577 10.24875/CIRU.20000916

[6] Rodriguez Colon R Park J J Boczar D Evaluating functional outcomes in reanimation surgery for chronic facial paralysis: a systematic review Plast Reconstr Surg Glob Open 2021 9 03 e3492 33758730 10.1097/GOX.0000000000003492 PMC7972661

[7] Terzis J K Noah M E Analysis of 100 cases of free-muscle transplantation for facial paralysis Plast Reconstr Surg 1997 99 07 1905 1921 9180714 10.1097/00006534-199706000-00016

[8] Terzis J K Tzafetta K “Babysitter” procedure with concomitant muscle transfer in facial paralysis Plast Reconstr Surg 2009 124 04 1142 1156 19935298 10.1097/PRS.0b013e3181b2b8bc

[9] Banks C A Bhama P K Park J Hadlock C R Hadlock T A Clinician-graded electronic facial paralysis assessment: the eFACE Plast Reconstr Surg 2015 136 02 223e 230e 10.1097/PRS.0000000000001447 26218397

[10] Banks C A Jowett N Azizzadeh B Worldwide testing of the eFACE facial nerve clinician-graded scale Plast Reconstr Surg 2017 139 02 491e 498e 10.1097/PRS.0000000000002954 28121888

[11] Mato-Patino T Sánchez-Cuadrado I Peñarrocha J Validation of the Spanish version of the Electronic Facial Palsy Assessment (eFACE) Eur Arch Otorhinolaryngol 2024 281 02 673 682 37535079 10.1007/s00405-023-08132-4 PMC10796419

[12] Cardenas-Mejia A Covarrubias-Ramirez J V Bello-Margolis A Rozen S Double innervated free functional muscle transfer for facial reanimation J Plast Surg Hand Surg 2015 49 03 183 188 25469588 10.3109/2000656X.2014.988218

[13] Rubio-Mainardi M S Palafox-Vidal D Cárdenas-Mejía A Caracterización electrofisiológica y análisis con la escala eFACE de la parálisis facial en el espectro óculo aurículo vertebral Cir Plást Iberolatinoam 2020 46 02 169 176

[14] Palafox D Rubio-Mainardi S Becerra R Cárdenas-Mejía A Validation of the clinician-graded electronic facial paralysis assessment Plast Reconstr Surg 2018 141 04 614e 615e 10.1097/PRS.0000000000004221 29596203

[15] Cardenas-Mejia A Palafox D Facial reanimation surgery in Möbius syndrome: experience from 76 cases from a tertiary referral hospital in Latin America Ann Chir Plast Esthet 2018 63 04 338 342 29153254 10.1016/j.anplas.2017.10.008

[16] Fukumoto-Inukai K A Palafox D Chávez-Serna E Facial reanimation in Moebius syndrome using bilateral free gracilis transfer: 1-stage versus 2-stage procedures Plast Reconstr Surg 2025 156 04 567e 575e 40100160 10.1097/PRS.0000000000012106

[17] Kim M J Kim H B Jeong W S Choi J W Kim Y K Oh T S Comparative study of 2 different innervation techniques in facial reanimation: cross-face nerve graft-innervated versus double-innervated free gracilis muscle transfer Ann Plast Surg 2020 84 02 188 195 31688275 10.1097/SAP.0000000000002034

[18] Biglioli F Frigerio A Rabbiosi D Brusati R Single-stage facial reanimation in the surgical treatment of unilateral established facial paralysis Plast Reconstr Surg 2009 124 01 124 133 19568051 10.1097/PRS.0b013e3181aa0e2b

[19] Tzafetta K Al-Hassani F Pinto-Lopes R Wade R G Ahmad Z Long-term outcomes of dual innervation in functional muscle transfers for facial palsy J Plast Reconstr Aesthet Surg 2021 74 10 2664 2673 33853750 10.1016/j.bjps.2021.03.007

[20] Kimura T Narita K Oyamada K Fine-tuning on AI-driven video analysis through machine learning: development of an automated evaluation tool of facial palsy Plast Reconstr Surg 2025 155 06 1071e 1081e 10.1097/PRS.0000000000011924 39688730

